# A case-series of patients with musculoskeletal conditions in an underserved community in Moca, Dominican Republic

**DOI:** 10.1186/s12998-019-0294-3

**Published:** 2020-02-04

**Authors:** Sophia da Silva-Oolup, Margareta Nordin, Paula Stern, Geoff Outerbridge, Pierre Côté

**Affiliations:** 1 Division of Graduate Education and Research Programs, Canadian Memorial Chiropractic College, 6100 Leslie St, Toronto, Ontario M2H 3J1 Canada; 2 Departments of Orthopedic Surgery and Environmental Medicine, New York University, New York, NY USA; 3 World Spine Care, Santa Ana, CA USA; 4 UOIT-CMCC Centre for the Study of Disability Prevention and Rehabilitation, Toronto, Ontario Canada; 5 Faculty of Health Sciences, University of Ontario Institute of Technology (UOIT), Oshawa, Canada; 6 Canada Research Chair in Disability Prevention and Rehabilitation, University of Ontario Institute of Technology (UOIT), Oshawa, Ontario Canada

**Keywords:** Musculoskeletal diseases, Spine, Dominican Republic, Developing countries, Chronic pain

## Abstract

**Purpose:**

To describe characteristics and activity limitations of new patients with musculoskeletal conditions presenting to the World Spine Care (WSC) clinic located in the underserved community of Moca, Dominican Republic.

**Methods:**

We conducted a prospective case series of consecutive adults between October 12 and December 5, 2015. A survey of valid and reliable measures including: Body pain diagram, Wong-Baker FACES® pain rating scale, Spine Functional Index (SFI), Lower Limb Functional Index (LLFI), Upper Limb Functional Index (ULFI) and the 12-item Short Form Health Survey (SF-12v2) was administered to collect socio-demographics, expectation of recovery, comorbidities, and self-reported health status data.

**Results:**

Forty-two patients (23 females and 19 males) were included. The most common primary complaint was lower back pain (40.5%; 17/42) and 57% (24/42) of individuals reported pain that interfered with their ability to function and engage in daily activities. Half of the patients presented with two complaints. Complaints were similar between genders. Most patients (64%; 27/42) reported chronic pain (> 6 months) and 97% (41/42) reported believing that they would recover. Twenty-one percent (9/42) self-reported being diagnosed with depression and/or anxiety at some point in their life. In addition, most (57%; 24/42) individuals reported below average physical and mental health related quality of life.

**Conclusions:**

This study is the first to describe characteristics of patients seeking care at the WSC clinic in Moca, Dominican Republic. Most patients attending the clinic suffer from persistent spine complaints that interfere with their ability to function and engage in daily activities. Nevertheless, the patients have positive expectations of recovery.

## Introduction

Musculoskeletal conditions such as low back and neck pain are leading causes of disability in developing nations [1–3]. In the Dominican Republic low back pain and other musculoskeletal conditions rank amongst the top 10 causes of years lived with disability [4]. The disability associated with these conditions affects individuals’ abilities to work, maintain financial independence and care for and support their families [5]. The burden of disease associated with these disabilities is defined as the impact of a health condition as measured by financial cost, morbidity and mortality [6]. The burden of musculoskeletal disorders are important contributors to the demands placed on the current Dominican health care system [3, 7].

With a population of 10.4 million [8] the Dominican Republic is one of the poorest Latin American developing nations. It is considered an upper-middle income emerging economy [9, 10] with 42% of its population living below the poverty line (a third of these live in extreme poverty). The proportion of the gross domestic product (GDP) expenditure spent on health care is 6.1% [11]; which is half of what is spent in developed nations [12]. The Dominican Republic has one of the highest (56%) out of pocket expenses for health care services in comparison to other Latin American and Caribbean nations [13]. Residents of the Dominican Republic have a life expectancy of 77 years and a healthy life expectancy of 66 years [14]. In contrast, Canadians have a life expectancy of 82 and a healthy life expectancy of 72 years [15].

The burden of disease attributable to musculoskeletal conditions has increased by 60% from 1990 to 2010 in developing nations globally [16]. In the Dominican Republic there has been a 21% increase in the years lived with disability due to low back and neck pain [16]. The Dominican Republic is poorly equipped to deal with the growing burden of musculoskeletal conditions due to the low availability of health care resources to manage musculoskeletal conditions [7]. A recent review completed by the Global Spine Care Initiative has identified that there has been a dramatic increase in the prevalence, impact and burden of spinal pain from 1990 to 2015. They identified that a call for action is necessary for high-quality research and funding to better understand the burden of spinal conditions on low and middle-income countries [17, 18].

World Spine Care (WSC) is a non-profit organization with a mission to improve the lives of individuals in underserved communities through sustainable, integrated evidence-based spine care. World Spine Care opened in Moca, Dominican Republic in November 2014 [19]. The Moca clinic is located in a rehabilitation center run by the Patronato Cibao de Rehabilitacion. The clinic is also located on-site behind the public hospital in Moca. The community of Moca is located in the north-central region of the Dominican Republic in the Espaillat province. The population of Moca is approximately 173,000 with 24,300 households [13]. The economy in Moca is agriculturally based, centered on the production of cacao, tobacco, coffee and sugarcane [20].

Little is known about the health care utilization for musculoskeletal conditions in the Dominican Republic. Therefore, the objective of our study was to describe the socio-demographic characteristics, health status, functional limitations and self-reported quality of life of adults presenting to a World Spine Care clinic located in Moca, Dominican Republic.

## Methods

We conducted a prospective case series of consecutive new patients presenting to the WSC clinic in Moca, Dominican Republic between October and December, 2015. A prospective case series is a small exploratory study that has been approved by an ethics board, in which participants enrolled provide informed consent to participate prior to data collection. The primary investigator was responsible for participant recruitment, data collection and obtaining consent. We defined cases as adults presenting to the WSC clinic in Moca for an initial assessment. To be included, cases had to be at least 18 years of age and fluent in Spanish or English. Participation in the study was voluntary; no compensation was provided for participation. All participants provided written informed consent and all information was kept confidential and anonymous. Participants were recruited between October 12, 2015 and December 5, 2015.

We collected data using a paper and pencil questionnaire that included five sections. The questionnaire, available in English and Spanish [21–33] was self-administered or completed with the assistance of a trained Spanish-native translator, the primary investigator or a family member. The following sections were included in the questionnaire:

1. WSC initial intake forms: Used to collect demographic characteristics, socioeconomic status, expectation of recovery (individuals were asked “do you think you will recover from your pain?”, general health, comorbidities and activities of daily living characteristics.
2. Body pain diagram: Used to measure self-perceived pain location and distribution. The body pain diagram has proven to be an adequate measure for assessing pain distribution and pain location with good reliability (k = 0.77–0.81) [21].
3. Wong-Baker FACES® pain rating scale: Used to measure self-perceived pain intensity. The measure shows a series of 6 faces that depict pain sensation from “no hurt” to “hurts worst”. The measure has shown adequate agreement with Visual Analogue Scale (*r* = 0.90; 95% CI = 0.86 to 0.93) and inter-rater reliability (ICC = 0.93) [22–25]. Appropriate permission was obtained for the use of this scale in the study.
4. Functional outcome measures (Spine Functional Index, Upper Limb Functional Index, Lower Limb Functional Index): Used to measure self-perceived functional ability. The specific functional index measures are a single page 25-items with a three-point Likert scale. A higher total score represents a higher level of functional disability. All items are summed for a maximum total score of 25. A percentage score (100-scale score) can be calculated and a lower percentage represents a higher level of functional disability. The recall period is within the last few days. These measures have been shown to be valid (*r* = 0.85–0.9) and reliable (ICC = 0.93–0.96) in both the English and Spanish languages [26–31]. If a patient presented with a primary complaint of low back pain, the Spine Functional Index was completed. If the participant presented with a primary complaint of knee pain, the Lower Limb Functional Index was given. If the primary complaint was shoulder pain, the Upper Limb Functional Index was given. If the participant presented with multiple complaints, multiple outcome measures were completed corresponding to the respective regions of complaint.
5. 12-item Short Form Health Survey (SF-12v2): We used the SF-12v2 to measure of health-related quality of life. Scores range from 0 to 100, with a higher score indicating better self-perceived health. The acute (1-week) recall SF-12v2 was used. Physical Component Summary (PCS) and Mental Component Summary (MCS) component scores were calculated. The SF-12v2 is a valid (correlated to the SF-36) (*r* = 0.95–0.96) and reliable (test-retest) (α = 0.75–0.82) measure that has been used widely to assess the impact of musculoskeletal diseases in various populations as well as in the Dominican Republic [32, 33]. The Spanish version of the SF-12v2 has been shown to have good reliability and validity [34]. Appropriate permission was obtained for the use of this scale in the study.

The primary investigator reviewed all surveys following completion and any missing data and/or clarifications were reviewed with the participant and the researcher.

All data was entered by the primary investigator into an electronic database on a daily basis. We verified the accuracy of the data by completing double data entry. We computed descriptive statistics to describe our series of cases. Specifically, we used proportions, means and standard deviations. We conducted all analyses using STATA v.13.1 for Windows. The Quality Metric Health Outcomes Scoring Software 2.0 was used to score the SF-12v2 data.

This study was approved by the Canadian Memorial Chiropractic College Research Ethics Board (#1504X03) and Consejo Nacional de Bioetica en Salud (CONABIOS) in the Dominican Republic (August 19, 2015).

## Results

### Sample characteristics

Forty-three patients presented to the clinic between October 12, 2015 and December 5, 2015. No patients refused to participate but one participant was excluded because of age (< 18 years old). The final sample included 42 participants. Twenty-six participants (62%) completed the questionnaire with assistance from a clinical assistant, the primary investigator and/or a family member. All of the surveys were completed in the Spanish language.

The age of participants ranged from 20 to 77 years old (mea*n* = 45 years; SD + 16.6). Fifty-five percent (23/42) were women; most participants were married (38%; 16/42) and 45% (19/42) had post-secondary education (university or college). Ninety-three percent (39/42) were able to read a newspaper.

### Occupation and lifestyle

All participants reported to be working in paid or unpaid work and 62% (26/42) of those described their work as physically demanding. Common occupations included housework (21%; 9/42) office work (19%; 8/42) and working in the service industry (12%; 5/42) and retirees (12%; 5/42) (Table 1).

**Table 1:** Baseline characteristics of patients attending the Moca clinic between October and December 2015

Demographic characteristics	Study sample *n* = 42 n (%)	Male *n* = 19 n(%)	Female *n* = 23 n(%)
Age group(yrs)			
18–24	5 (11.9)	3(15.8)	2(8.7)
25–34	7 (16.6)	4 (21)	3 (13)
35–44	11 (26.2)	4 (21)	7 (30.4)
45–54	8 (19)	4 (21)	4 (17.4)
55–64	4 (9.5)	3(15.8)	1 (4.3)
65–74	4 (9.5)	1 (5.3)	3 (13)
75+	3 (7.1)	0	3 (13)
Occupation			
Retired	5(11.9)	3(15.8)	2(8.7)
Unemployed	1(2.4)	0	1 (4.3)
Student	3 (7.1)	2(10.5)	1 (4.3)
Farmer	1 (2.4)	1 (5.3)	0
Office worker	8(19)	2(10.5)	6 (26.1)
Labourer	1(2.4)	1 (5.3)	0
Housework	9 (21.4)	0	9 (39.1)
Teacher	1(2.4)	1 (5.3)	0
Health care	3 (7.1)	1 (5.3)	2(8.7)
Other professional	1(2.4)	1 (5.3)	0
Service	5 (11.9)	4 (21)	1 (4.3)
Other	4 (9.5)	3(15.8)	1 (4.3)
Spend most of time doing			
Manual labour	9 (21.4)	4 (21)	5(21.7)
Driving	2(4.76)	1 (5.3)	1(4.3)
Sitting	14(33.3)	5(26.3)	9(39.1)
Standing	5(11.9)	3(15.8)	2(8.7)
Walking	8(19)	2(10.5)	6(26.1)
Cycling	1(2.4)	1 (5.3)	0
Other	3(7.1)	3(15.8)	0
Participate in walking			
Never	2(4.8)	0	2(8.7)
Sometimes	19(45.2)	7(36.8)	12(52.2)
Regularly	18 (42.9)	11(57.9)	7(30.4)
Pain prevents	3(7.1)	1 (5.3)	2(8.7)
Belief in recovery	41 (97.6)	19 (100)	22 (95.7)

*Yrs* years

Most participants (79%; 33/42) previously consulted a health care professional for their complaints. Five percent of participants (2/42) were current smokers and 19% (8/42) had smoked in the past. Fifty-seven percent (24/42) reported that they currently consume alcohol. This was equal between genders. Twenty-one percent (4/19) of males engaged in sport regularly while only 4% (1/23) of females reported engaging in sport on a regular basis (Table 1).

### Pain and regions of complaint

Half of the participants (21/42) reported two musculoskeletal complaints and 7% (3/42) presented with three complaints. Most primary complaints were lower back pain (40.5%; 17/42) upper back pain (14%; 6/42), neck pain (12%; 5/42) and shoulder pain (12%; 5/42) (Table 2). The mean pain intensity was 5.3/10 (SD + 2.78). Thirty-three percent of participants (14/42) reported worsening of their symptoms since onset, 36% (15/42) reported their pain was the “same” and 31% (13/42) reported their pain had improved since its onset. Most participants (64%; 27/42) reported the duration of pain of their primary complaint had been present for more than a year. Nineteen percent (8/42) of participants reported current complaints due to a work-related injury. Most participants (57%; 24/42) reported pain that interfered with their daily activities. Sixty-four percent (27/42) of participants self-reported being diagnosed with chronic pain (> 6 months in duration) and chronic pain was more common in females (70%; 16/23) than males (58%;11/19).

**Table 2:** Sex-specific frequency distribution of pain and regions of complaint

Variable	Study Sample *N* = 42 n (%)	Male *n* = 19 n(%)	Female *n* = 23 n(%)
Number/Rank of complaints			
One/Primary	41 (97.6)	19 (100)	22 (95.7)
Two/Secondary	21 (50)	8 (42.1)	13 (56.5)
Three/Tertiary	3 (7.1)	2(10.5)	1 (4.3)
Primary regions of pain			
No pain	1(2.4)	0	1 (4.3)
Neck	5(11.9)	3(15.8)	2 (8.7)
Shoulder	5(11.9)	4 (21)	1 (4.3)
Arm	1(2.4)	0	1 (4.3)
Upper back	6(14.3)	1 (5.3)	5 (21.7)
Lower back	17(40.5)	7 (36.8)	10 (43.5)
Hip	3(7.1)	1 (5.3)	2 (8.7)
Thigh	1(2.4)	1 (5.3)	0
Knee	1(2.4)	1 (5.3)	0
Ankle	1(2.4)	1 (5.3)	0
Foot	1(2.4)	0	1 (4.3)
Secondary regions of pain			
No pain	21 (50)	11 (57.9)	10 (43.5)
Neck	2(4.8)	1 (5.3)	1 (4.3)
Elbow	1(2.4)	1 (5.3)	0
Arm	2(4.8)	1 (5.3)	1 (4.3)
Upper back	3(7.1)	1 (5.3)	2 (8.7)
Lower back	7(16.6)	1 (5.3)	6 (26)
Knee	2(4.76)	1 (5.3)	1 (4.3)
Foot	2(4.76)	0	2 (8.7)
Legs	1(2.4)	1 (5.3)	0
Upper extremity	1(2.4)	1 (5.3)	0
Tertiary regions of pain			
No pain	39 (92.9)	17 (89.5)	22 (95.7)
Neck	1(2.4)	1 (5.3)	0
Shoulder	1(2.4)	0	1 (4.3)
Lower back	1(2.4)	1 (5.3)	0
Pain duration			
< month	1 (2.4)	0	1(4.3)
1–3 months	9 (21.4)	6(31.6)	3(13)
4–6 months	3 (7.1)	1 (5.3)	2(8.7)
7–12 months	2 (4.8)	1 (5.3)	1(4.3)
> 12 months	27 (64.3)	11(57.9)	16(69.6)

> greater than, *<* less than

### Functional impact and daily activities

Nineteen percent of participants (8/42) reported that their current pain prevented them from engaging in sport; this was equal between genders. Participants reported spending the majority of their day sitting (33%; 14/42), followed by engaging in manual labour (21%; 9/42), walking (19%; 8/42) and standing (12%; 5/42). Seven percent of participants (3/42) reported that their current pain prevented them from walking (Table 1).

The mean functional index scores were 12.0/25 (SD + 6.3) for spinal complaints 12.6/25 (SD = + 8.4) for upper limb complaints and 12.7/25 (SD + 6.4), for lower limb complaints. Functional index measures were also stratified based on rank of complaint. These measures indicated that individuals had a moderate (50%) level of functional disability that impacted their daily lives (Table 3).

**Table 3:** Functional outcome measures

Variable	Total score Mean (SD)	Percentage score Mean (SD)
Spine functional index		
Total score (*n* = 42)	12.03 (6.4)	52.47 (25.83)
Primary rank (*n* = 28)	10.73 (5.45)	57.79(22.47)
Secondary rank (*n* = 12)	13.42(6.87)	46.33(27.47)
Tertiary rank (*n* = 2)	23(0.71)	8(2.83)
Upper limb functional index		
Total score (*n* = 11)	12.64 (8.37)	49.45 (33.48)
Primary rank (*n* = 6)	14.17(10.17)	43.33(40.69)
Secondary rank (*n* = 4)	8.38(3.4)	66.5(13.6)
Tertiary rank (*n* = 1)	20.5 (0)	18 (0)
Lower limb functional index		
Total score (*n* = 12)	12.73 (6.44)	49.09 (25.79)
Primary rank (*n* = 7)	10.64 (6.34)	57.43(25.37)
Secondary rank (*n* = 5)	14.3(6.65)	42.8 (26.6)
Tertiary rank (*n* = 0)	0	0

Total Score Range: 0 to 25; a higher score indicates a higher level of functional disability

Percentage Score: 0–100%; a lower percentage represents a higher level of disability

*SD* standard deviation

### General health and comorbidities

Participants perceived their self-reported physical and mental health related quality of life to be lower than the US adult norm of 50. The mean physical component score (PCS) for females was 38.4 (SD + 10.2) in comparison to males with a mean of 42.7 (SD + 11.8). The mean mental component score was 46.8 (SD + 11.0), which was similar between genders. One in five participants (21.4%; 9/42) self-reported having been diagnosed with depression and/or anxiety at some point in their lives.

Most participants (97.6%; 41/42) reported that they expected to recover from their current health conditions (Table 1). Forty-one percent (17/42) reported experiencing joint problems, 31% (13/42) reported hypertension and 14%(6/42) reported being diabetic. In addition, 29% (12/42) and 19% (8/42) of participants reported having lung or heart conditions with asthma (19%; 8/42) being the most common (Table 4).

**Table 4:** Sex-specific frequency distribution of comorbidities

Variable	Study sample *N* = 42 n (%)	Men *n* = 19 n(%)	Women *n* = 23 n(%)
Osteoporosis	5 (11.9)	1 (5.3)	4 (17.4)
Joint problems	17 (40.5)	7 (36.8)	10 (43.5)
Diabetes	6(14.3)	2(10.5)	4 (17.4)
Lung condition	12(28.6)	4 (21)	8 (34.8)
Heart condition	8 (19)	3(15.8)	5 (21.7)
Hypertension	13 (31)	6 (31.6)	7 (30.4)
Stroke	2 (5)	1 (5.3)	1 (4.3)
Depression-anxiety	9 (21.4)	3(15.8)	6 (26)
Chronic pain	27(64.3)	11(57.9)	16(69.6)

Lung Condition: Pneumonia/Asthma

Heart Condition: Unknown/Angina/Tachycardia/Atherosclerosis/Heart Disease

Chronic Pain: greater than 6 months

Joint Problems: Participants were asked: Have you ever had any other health problems related to the joints?

## Discussion

We described the characteristics of patients who presented to the WSC clinic in the rural underserved community of Moca, Dominican Republic between October 12, 2015 and December 5, 2015. Our study suggests that most individuals presenting to the WSC clinic in Moca complained of persistent spinal complaints. Most participants reported complaints that interfered with their abilities to engage in activities of daily living. However, all individuals indicated that they continued working either in paid or unpaid occupations. Moreover, most individuals expected to recover.

The current study’s findings are consistent with the findings of the 2013 Global Burden of Disease Study which identified that in the Dominican Republic major depressive disorder (#1), low back pain (#2), anxiety (#4), neck pain (#8) and other musculoskeletal conditions (#10) were within the top 10 causes of years lived with disability [35]. Our study is also consistent with the findings of the recent systematic review conducted by the Global Spine Care initiative which identified that spinal disorders primarily lower back and neck pain are common in various populations around the world; and that lower back pain is more common than neck pain [18].

In 2009 a survey conducted in three rural underserved villages in the Dominican Republic found that hypertension (36%), diabetes (15%) and asthma (14%) were common in these populations [36]. Our results agree with these figures. Furthermore, 70% of the sample from the study by Caban-Martinez et al. reported fair to poor perceived general health status; which is similar to the current study where the majority (57%) of individuals reported low health related quality of life.

In a previous study conducted in the Monti Cristi region of the Dominican Republic, 18.6% of individuals reported experiencing general pain (back pain, nonspecific headaches, general pain, non-specific muscle strain and osteoarthritis). In addition, general pain was the most frequently reported symptom [37]. The current study is similar to this larger study in which the majority of individuals reported experiencing musculoskeletal complaints.

The findings from these two prior studies [36, 37] combined with ours are helpful to generate hypotheses of the association between musculoskeletal conditions and disability in low- and middle-income countries. It is important for clinicians and researchers to understand the associations between these conditions to ensure that appropriate educational, patient referral networks and management strategies can be implemented at the WSC clinic. This could potentially not only improve patient health and well-being but also decrease disability and the burden of non-communicable diseases, in particular musculoskeletal conditions.

Our study agrees with a previous study which assessed musculoskeletal disorders in the medically underserved regions of Peru, Ecuador, Argentina and Vietnam in which participants were sampled during brief medical outreach mission trips. In those countries studied, the prevalence of musculoskeletal symptoms was high. The South American patients reported experiencing acute pain most commonly in the lower back (44.7%) followed by the upper back (46.6%). The Vietnamese patients reported experiencing acute pain most commonly in the knees (44.7%) followed by the upper back (35.0%). In addition, for patients in both samples, acute pain was associated with chronic pain in the same location for all body parts (*p* < 0.1) [38]. Our study also suggests that the impact of musculoskeletal conditions may be similar among different underserved developing nations.

The 2017 Global Burdens of Disease Study identified that in Canada low back pain (#1), diabetes (#3), depressive disorders (#4), other musculoskeletal conditions (#6) and neck pain (#10) were within the top 10 causes of years lived with disability [39]. The findings of our study completed within a developing nation are quite similar to those identified in the developed nation of Canada. This suggests that the current evidence available from developed nations in regards to the management of musculoskeletal conditions could be applied to those living in developing nations; however further high quality research should be conducted.

### Strengths and limitations

Our study had strengths. First, a consecutive sample of participants were recruited. Second, no participants refused to participate in the study. Third, the primary investigator reviewed all questionnaire for completeness. Our study also has limitations. First, the psychometric properties of our questionnaire were not previously tested for use in our population. However, the functional outcome measures have appropriate reliability and validity in the Spanish language [27, 29, 31]. Second, 62% of participants required assistance (translator, family or primary investigator) to complete study surveys. This may have affected participant’s responses. Third, all data was based on subjective self-report and it was not feasible to access participant’s medical records to confirm and/or refute the self-report of chronic diseases and patient responses. Fourth, we recruited 42 patients within a two-month period. A longer recruitment period would have led to a larger sample size and possibly provided a more comprehensive description of patients attending the clinic.

### Challenges

Our study highlights the challenges of conducting research in an emerging nation. Specifically, the process involved in obtaining approval from the Consejo Nacional de Bioetica en Salud (CONABIOS) Ethics Review Board was complex and lengthy. We needed to collaborate with an intermediary representative from the Dominican Republic who communicated with the Ministry of Health Board on behalf of the research team. This process led to significant delays in initiating the research.

The Dominican culture is different from the culture of western countries. Specifically, participants are not accustomed to the lengthy questionnaires needed to conduct clinical research. This typically resulted in participants becoming fatigued with paperwork and requiring support to ensure appropriate completion. Moreover, the relaxed attitude towards schedules often had patients attending the clinic at the times that suited them without prior notifications. This is concerning because these factors can affect participant recruitment, and appropriate study completion timeframes.

In future research, investigators should ensure that an appropriate time (6–9 months) is allocated to guarantee ethics approval. In addition, researchers should streamline paperwork, ensure all paperwork is organized, and can be completed and checked efficiently by research staff and patients. Individuals should be cognizant of the burden that extensive paperwork places on clinical support staff and patients and work with a dedicated clinical assistant. To optimize participant recruitment, data collection and study completion, future researchers should ensure that appropriate communication regarding travels dates, clinic hours and national holidays occurs between all involved organizations.

## Conclusions

Our study was the first to describe characteristics of patients seeking care at the WSC clinic in Moca, Dominican Republic. Our results suggest that most individuals seek care for primarily chronic spine complaints that interfere with their ability to function and engage in daily activities.

## Data Availability

The data used and/or analysed during the current study are available from the corresponding author on reasonable request.
